# The gender difference of snore distribution and increased tendency to snore in women with menopausal syndrome: a general population study

**DOI:** 10.1007/s11325-016-1447-4

**Published:** 2016-12-23

**Authors:** Li-Pang Chuang, Shih-Wei Lin, Li-Ang Lee, Hsueh-Yu Li, Chih-Hao Chang, Kuo-Chin Kao, Li-Fu Li, Chung-Chi Huang, Cheng-Ta Yang, Ning-Hung Chen

**Affiliations:** 1 Department of Pulmonary and Critical Care Medicine, Sleep Center, Chang Gung Memorial Hospital, 5, Fushing Street, Gueishan Shiang, Taoyuan, Taiwan; 2 School of Medicine, Chang Gung University, 259 Wen-Hwa 1st Road, Gueishan Shiang, Taoyuan, Taiwan; 3 Department of Otolaryngology, Sleep Center, Chang Gung Memorial Hospital, 5, Fushing Street, Gueishan Shiang, Taoyuan, Taiwan

**Keywords:** Snore, Gender, Women, Menopausal syndrome, Hypertension

## Abstract

**Purpose:**

Sleep-disordered breathing (SDB) is a prevalent disorder with a major impact in women, especially postmenopausal women. However, few studies have investigated the prevalence of a specific SDB, snoring, among women especially those with menopausal syndrome.

**Methods:**

Computer-assisted telephone interviews were conducted in Taiwan. Adults over 20 years of age were interviewed. The number of successful interviews was calculated based on the population prior to the study. Demographic data and information about snoring, menopausal syndrome, and medical conditions were obtained.

**Results:**

In total, 3624 adults, 1473 males and 2151 females, completed the interviews. Both men and women shows an increase in snoring until age 50 to 59 years, followed by a decline in snoring that is less steep among women. The prevalence of snoring increased significantly in females after age 50 years, which is the mean menopausal age in our country (*p* < 0.05). After adjusting for age, body mass index, and other major diseases, the percentage of women with snoring was significantly higher among those with menopausal syndrome than those without menopausal syndrome (*p* = 0.021, odds ratio = 1.629).

**Conclusions:**

This population-based study revealed different snoring percentages among men and women and diminishing differences in the older population. Additionally, the percentage of women with snoring was increased among those women who were older than 50 years and those with menopausal syndrome.

## Introduction

Sleep-disordered breathing (SDB) is a prevalent disorder associated with daytime sleepiness, decreased ability to concentrate, and increased vascular events, such as hypertension, heart attack, and stroke [1]. Snoring can be a symptom of obstructive sleep apnea (OSA), which is the most severe form of SDB, and may be a precursor to OSA [2]. The presence of snoring implies an elevated upper airway resistance with limited inspiratory flow [3]. Such episodes of flow limitation are frequently terminated by an arousal (i.e., respiratory effort-related arousals), which leads to OSA. Early studies have shown that even trivial snoring may be the first step in a continuum leading to a full-blown OSA syndrome [4]. Although snoring is not synonymous with SDB, isolated studies imply that snoring may be a risk factor for certain medical conditions [5].

Menopause defines the period in a woman’s life when menstruation stops and the body goes through changes that no longer allow pregnancy [6]. Menopausal syndrome has many symptoms, including hot flashes, night sweats, vaginal dryness, palpitations, headaches, insomnia, lack of energy, and depression [7]. One of the most common symptoms, hot flashes, is related to the changing hormone levels [8]. The exact etiology and pathogenesis of hot flashes have not yet been fully understood, but it may be due to a change in the ability of the hypothalamus to regulate body temperature [9].

Literature already exists about the increase in snoring and SDB among menopausal women [10–14]. However, few data have been published about the relationship between snoring and menopausal syndrome. The aim of this population-based study was to clarify the gender difference of snore distribution and the association between snoring and the menopausal syndrome.

## Materials and methods

### Subjects

Adults over 20 years of age currently residing in Taiwan were eligible for this study. The number of people to be interviewed was calculated according to the estimated prevalence in previous reports and the population distributions in each county [15]. From October 25, 2006, to November 6, 2006, 11,649 individuals selected randomly from Taiwan’s national telephone directory were contacted using a computer-assisted telephone interviewing system (CATI) [16]. A total of 3862 (33.2%) people refused to be interviewed for any reason, and 4163 (35.7%) people did not complete the interview due to age less than 20 years, language barriers, communication difficulties, and poor telephone/cell phone quality. In total, 3624 (31.1%) people successfully completed the interview during the investigational period.

### Operation of CATI

The CATI system is a well-established method of conducting telephone-based interviews with the assistance of a computer. This popular methodology involves a telephone interviewer reading from a computer-based question and typing in the answers as they are reported. Participants were interviewed by 40 well-trained telephone investigators. The precise operation of CATI in the current study is similar to another population-based study [17]. Individual telephone numbers were selected randomly from Taiwan’s national telephone directory and were dialed digitally. Oral informed consent was obtained before starting the procedure, and the study was approved by the Institutional Review Board of Chang Gung Memorial Hospital.

### Questions on the CATI

Questions at the prompts during the telephone interview obtained data on snoring, menopausal syndrome, major medical conditions, and demographic information. Participants were asked, “Do you or your family members hear you snore when you sleep?” and (for women) “Do you have symptoms of menopause syndrome such as hot flashes?” Other questions addressed major medical conditions in the past year, such as hypertension (defined as a self-reported diagnosis of hypertension made by a physician or an individual under treatment for hypertension), cardiovascular disease (defined by self-reported angina or myocardial infarction/heart attack), diabetes mellitus, arthritis, respiratory disease (e.g., chronic bronchitis, asthma, emphysema), anemia, and mental illness (e.g., depression or manic-depressive disorder). Demographic data, such as age, gender, weight, height, and body mass index (BMI), were obtained.

### Statistical analysis

The chi-squared test was applied to compare categorical data and independent samples. *T* tests were applied to compare the mean values of the two groups. Multivariate logistic regression was used to test the relationship and determine the odds ratio of snoring with age, BMI, major medical conditions, and menopausal syndrome. Data were presented as mean ± SD. All statistical tests utilized the SPSS software (SPSS Institute, NY, USA), and a value of *p* < 0.05 considered statistically significant.

## Results

**Fig. 1:**
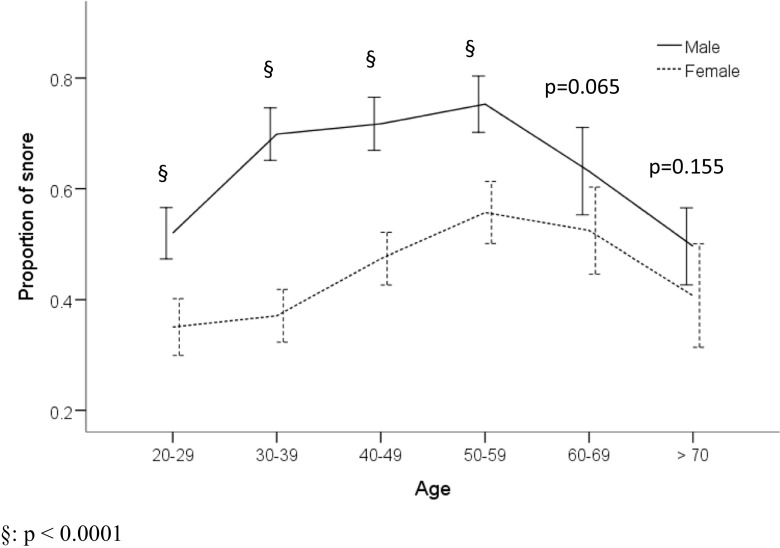
The distribution of snoring among different age groups in men and women. The proportion of men and women remains statistically significant in each age group up to age 50 to 59 years; however, the statistical significance disappears in the older age groups

Figure 1 shows the distribution of snoring among different age groups of men and women. A trend toward increased snoring was observed in men and women until the age group of 50 to 59 years. The proportion of both men and women who snore decreases after age 59, but the women experience a less steep decline compared to the men. The proportion of men and women who snore remains statistically significant in each age group up to age 50 to 59 years; however, the statistical significance disappears in the older age groups (*p* = 0.065 and *p* = 0.155 for ages 60 to 69 and ≥70 years, respectively).

**Fig. 2:**
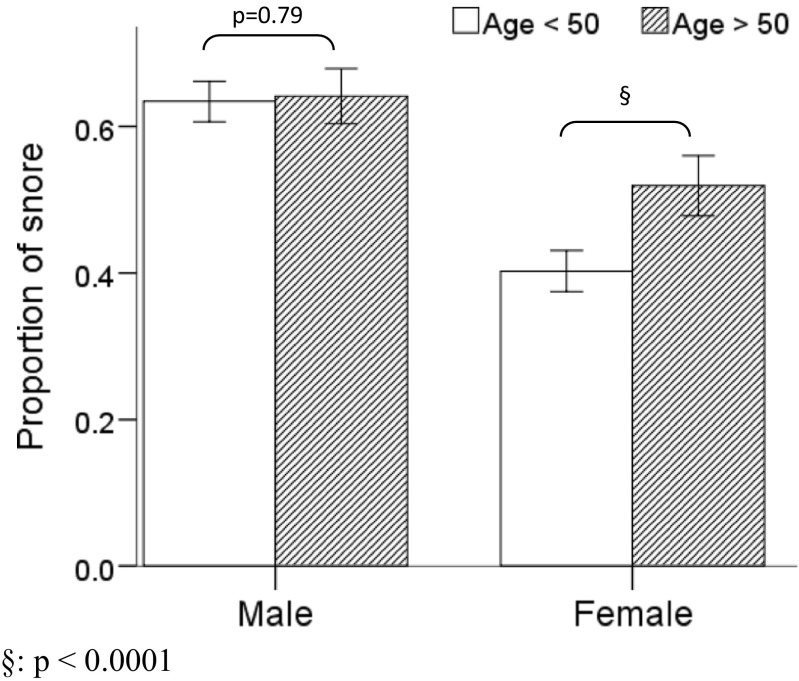
The prevalence of snoring in men and women before and after age of 50. The proportion of snoring was equal in men before and after the age of 50. However, among women, the proportion of snoring increases with statistical significance after the age of 50 (*p* < 0.0001)

The studied population was divided at age 50 years, which is the mean menopausal age in our country [18]. The proportion of snoring was equal in men before and after the age of 50. However, among women, the proportion of snoring increases with statistical significance after the age of 50 (*p* < 0.0001, Fig. 2).

To see the relationship between menopausal syndrome and snoring, the group of women 50 to 59 years of age, which is the highest prevalence of menopausal syndrome and also the peak snoring among our female population, was selected for further analysis. Table 1 demonstrates the characteristics of the female population between 50 and 59 years of age (*n* = 550). Among them, 170 women (31%) had menopausal syndrome. The mean age and BMI between women with menopausal syndrome and those without menopausal syndrome were not statistically different. The presence of cardiovascular disease and anemia were statistically significant (*p* < 0.001 and *p* < 0.008, respectively). The proportion of women with snoring was higher and statistical significant among women with menopausal syndrome (*p* = 0.016; Table 1).

**Table 1:** Characteristics of women 50 to 59 years of age with/without menopausal syndrome

	Menopausal syndrome		
	With (*n* = 170)	Without (*n* = 380)	*p* value
Age (yr)	53.7 ± 2.5	53.7 ± 2.9	0.959
BMI (kg/m^2^)	23.4 ± 3.2	23.7 ± 3.2	0.402
Snore	108 (63.5%)	196 (51.6%)	0.016
Hypertension	25 (14.5%)	76 (20.0%)	0.153
Cardiovascular disease	28 (16.3%)	14 (3.7%)	<0.001
Diabetes mellitus	10 (5.8%)	26 (6.8%)	0.714
Arthritis	35 (20.3%)	56 (14.7%)	0.105
Respiratory disease	18 (10.5%)	22 (5.8%)	0.075
Anemia	43 (25.0%)	59 (15.5%)	0.008
Mental illness	9 (5.2%)	17 (4.5%)	0.672

*BMI* body mass index

A multivariate logistic regression model adjusted for age, BMI, and major medical conditions was utilized to determine the odds ratio for various factors associated with snoring (Table 2). After adjusting for the aforementioned variables, menopausal syndrome was identified as a significant risk factor for snoring (*p* = 0.021; odds ratio = 1.629). Individuals with a higher BMI and hypertension were also more likely to snore (*p* < 0.001; odds ratio = 1.141 and *p* = 0.027; odds ratio = 1.784, respectively).

**Table 2:** Adjusted odds ratio for the factors associated with snoring among women aged 50 to 59 years

	*p* value	Odds ratio	95% Confidence interval
Menopausal syndrome	0.021	1.629	(1.077, 2.465)
Age	0.769	0.990	(0.925, 1.059)
BMI	<0.001	1.141	(1.069, 1.217)
Hypertension	0.027	1.784	(1.067, 2.983)
Cardiovascular disease	0.750	1.138	(0.514, 2.520)
Diabetes mellitus	0.979	0.989	(0.449, 2.178)
Arthritis	0.258	1.362	(0.797, 2.329)
Respiratory disease	0.922	0.962	(0.441, 2.097)
Anemia	0.081	1.555	(0.948, 2.550)
Mental illness	0.769	0.990	(0.925, 1.059)

*BMI* body mass index

## Discussion

This population-based study investigated the distribution of snoring across the age ranges among adult residents in Taiwan by CATI. We found a different distribution of snoring among men and women; however, the proportion of men and women who snored peaked at 50 to 59 years of age. Females experienced a less steep drop in snoring than men beyond 59 years of age. It has been fairly well established that the prevalence of sleep apnea increases with age in both sexes. The prevalence of sleep apnea peaks in clinical populations around 55 years for men and 65 years for women [10]. Our data showed the same trend; however, women did not show a later peak in snoring as the previous study suggested. This finding may be explained by the different ethnic groups and/or different targets, such as snoring or sleep apnea, that were studied.

Our data revealed a generally lower snoring prevalence in the female population compared to male population for all age groups; however, the difference was less significant in older age groups. The role that estrogen plays in SDB is an ongoing area of research. Menopause normally occurs in women between ages 45 and 55 [6]. Increases in SDB, including snoring and sleep apnea, have been observed in postmenopausal women [10–14]. Increasing central obesity from the drop in estrogen associated with menopause is likely the main factor responsible for the increased prevalence of sleep apnea in the postmenopausal state [19]. In fact, SDB prevalence rates were lower in postmenopausal women who were taking hormonal replacement therapy (HRT) [11]. This increase in SDB was also documented in other Asian countries [20]. The prevalence of snoring has been reported as higher in postmenopausal women and women with irregular menstruation within the Chinese population [21]. Our data supported the same result with an increased snoring proportion of women older than 50 years old, which is the mean menopausal age in our country [18].

Sleep disturbances from menopause are associated with hot flashes, mood disorders, and increased SDB [7, 8]. Hot flashes (defined as feelings of intense heat over the trunk and face, flushing of the skin, and sweating) occur in 50 to 80% of menopausal women as a result of the decrease in ovarian hormones [22]. An increase in pulsatile release of gonadotropin-releasing hormone from the hypothalamus is believed to trigger the hot flashes by affecting the adjacent temperature-regulating area of the brain [9]. It is generally believed that when hot flashes occur at night, the resultant sweating and insomnia lead to fatigue the following day. However, Freedman and Roehrs mentioned that hot flashes did not lead to sleep disturbances in symptomatic menopausal women and concluded that previous reports of increased sleep disturbance at menopause may be due to other sleep disorders such as sleep apnea [23]. Also, another study conducted with polysomnography showed that menopausal women with hot flashes experienced more restless leg complaints [13]. Our data supported the previous finding and demonstrated an increase in the proportion of women who snored among those with the menopausal syndrome, independent of either age or BMI.

The precise role of estrogen in the pathogenesis of menopausal symptom is not quite clear. Although endogenous estrogen levels do not differ substantially between postmenopausal women who have hot flashes and those who do not have them, the use of HRT with estrogen has been documented to markedly improve hot flashes [24]. Low levels of female sex hormones, including estradiol and progesterone, are associated with an increased probability for SDB in women, and even estrogen monotherapy was associated with a significant reduction in OSA severity [25]. As previous reports have indicated, sex hormones can alter respiratory control through several nuclei and receptors in the carotid body and brain stem, and progesterone levels have been reported to affect pharyngeal dilator muscle activity [26, 27]. Thus, the possible mechanism related to the increased snoring among women with menopausal syndrome may be explained by sex hormone deficiencies.

Women at high risk for SDB also more frequently reported arterial hypertension or heart disease compared to women at low risk for SDB, indicating a possible relationship between SDB and cardiovascular morbidity [28]. A population-based study reported that HRT with progestin and estrogen was effective in lowering blood pressure in hypertensive women with menopausal syndrome [29]. Our study also identified that women with menopausal syndrome not only experienced an increase in snoring but were also more likely to have hypertension, which may not be related to age or other medical conditions.

Our study demonstrated the increased prevalence of snoring among women with menopausal syndrome. However, some limitations should be mentioned. First, data about the utilization of HRT were not collected. Although the HRT prescription rate is less than 7% in our country, the lack of HRT data may cause some misinterpretations in our result [30]. Additionally, the question about snoring in CATI was not specific enough to gather information solely about the habitual snorer, which is the key symptom of obstructive sleep apnea. We suggest polysomnography be conducted to more accurately document SDB in women with menopausal syndrome. Finally, not high CATI response rate was noted in our study, although the number of people to be interviewed was calculated according to the estimated prevalence in previous reports and the population distributions in each county of Taiwan, around 30% CATI response rate was accepted for other study especially those nationwide survey [31].

In conclusion, this population-based study revealed different proportions of men and women who snored across difference age distributions; however, these numbers narrowed in the older aged populations. After adjusting for age, BMI, and other medical conditions, the proportion of women with snoring was increased among those women with menopausal syndrome.

## References

[1] White DP (2006). Sleep apnea. Proc Am Thorac Soc.

[2] Collop NA (2005). Obstructive sleep apnea syndromes. Semin Respir Crit Care Med.

[3] Stoohs RA, Knaack L, Blum HC, Janicki J, Hohenhorst W (2008). Differences in clinical features of upper airway resistance syndrome, primary snoring, and obstructive sleep apnea/hypopnea syndrome. Sleep Med.

[4] Lugaresi E, Cirignotta F, Coccagna G, Piana C (1980). Some epidemiological data on snoring and cardiocirculatory disturbances. Sleep.

[5] Valham F, Stegmayr B, Eriksson M, Hagg E, Lindberg E, Franklin KA (2009). Snoring and witnessed sleep apnea is related to diabetes mellitus in women. Sleep Med.

[6] Col NF, Fairfield KM, Ewan-Whyte C, Miller H (2009) In the clinic. Menopause. Ann Intern Med 150(7):ITC4-1-15; quiz ITC14-16 10.7326/0003-4819-150-7-200904070-01004 19349628

[7] Research on the menopause in the 1990s. Report of a WHO Scientific Group (1996). World Health Organ Tech Rep Ser 866:1-107 8942292

[8] Andrikoula M, Prelevic G (2009). Menopausal hot flushes revisited. Climacteric.

[9] Spetz AC, Zetterlund EL, Varenhorst E, Hammar M (2003) Incidence and management of hot flashes in prostate cancer. J Support Oncol 1(4):263–266, 269–270, 272–263; discussion 267–268, 271–262 15334868

[10] Bixler EO, Vgontzas AN, Lin HM, Ten Have T, Rein J, Vela-Bueno A, Kales A (2001). Prevalence of sleep-disordered breathing in women: effects of gender. Am J Respir Crit Care Med.

[11] Young T, Finn L, Austin D, Peterson A (2003). Menopausal status and sleep-disordered breathing in the Wisconsin Sleep Cohort Study. Am J Respir Crit Care Med.

[12] Freedman RR, Roehrs TA (2007). Sleep disturbance in menopause. Menopause.

[13] Hachul H, Andersen ML, Bittencourt LR, Santos-Silva R, Conway SG, Tufik S (2010). Does the reproductive cycle influence sleep patterns in women with sleep complaints?. Climacteric.

[14] Hung HC, FH L, HY O, JS W, Yang YC, Chang CJ (2014). Menopause is associated with self-reported poor sleep quality in women without vasomotor symptoms. Menopause.

[15] Chuang LP, Hsu SC, Lin SW, Ko WS, Chen NH, Tsai YH (2008). Prevalence of snoring and witnessed apnea in Taiwanese adults. Chang Gung Med J.

[16] Kirk M, Tribe I, Givney R, Raupach J, Stafford R (2006). Computer-assisted telephone interview techniques. Emerg Infect Dis.

[17] Chen NH, Chuang LP, Yang CT, Kushida CA, Hsu SC, Wang PC, Lin SW, Chou YT, Chen RS, Li HY, Lai SC (2010). The prevalence of restless legs syndrome in Taiwanese adults. Psychiatry Clin Neurosci.

[18] Chang C, Chow SN, Hu Y (1995). Age of menopause of Chinese women in Taiwan. Int J Gynaecol Obstet.

[19] Fogel RB, Malhotra A, Pillar G, Pittman SD, Dunaif A, White DP (2001). Increased prevalence of obstructive sleep apnea syndrome in obese women with polycystic ovary syndrome. J Clin Endocrinol Metab.

[20] Kim J, In K, You S, Kang K, Shim J, Lee S, Lee J, Park C, Shin C (2004). Prevalence of sleep-disordered breathing in middle-aged Korean men and women. Am J Respir Crit Care Med.

[21] Ip MS, Lam B, Tang LC, Lauder IJ, Ip TY, Lam WK (2004). A community study of sleep-disordered breathing in middle-aged Chinese women in Hong Kong: prevalence and gender differences. Chest.

[22] Mohyi D, Tabassi K, Simon J (1997). Differential diagnosis of hot flashes. Maturitas.

[23] Freedman RR, Roehrs TA (2004). Lack of sleep disturbance from menopausal hot flashes. Fertil Steril.

[24] Randolph JF, Sowers M, Bondarenko IV, Harlow SD, Luborsky JL, Little RJ (2004). Change in estradiol and follicle-stimulating hormone across the early menopausal transition: effects of ethnicity and age. J Clin Endocrinol Metab.

[25] Manber R, Kuo TF, Cataldo N, Colrain IM (2003). The effects of hormone replacement therapy on sleep-disordered breathing in postmenopausal women: a pilot study. Sleep.

[26] Behan M, Zabka AG, Thomas CF, Mitchell GS (2003). Sex steroid hormones and the neural control of breathing. Respir Physiol Neurobiol.

[27] Popovic RM, White DP (1998). Upper airway muscle activity in normal women: influence of hormonal status. J Appl Physiol.

[28] Young T, Finn L, Peppard PE, Szklo-Coxe M, Austin D, Nieto FJ, Stubbs R, Hla KM (2008). Sleep disordered breathing and mortality: eighteen-year follow-up of the Wisconsin sleep cohort. Sleep.

[29] Amoroso A, Garzia P, Ferri GM, Clementia C, Battaglia T, Clemenzia G (1996). Hypertension and menopausal syndrome: effects of hormone replacement therapy and antihypertensive drugs. Riv Eur Sci Med Farmacol.

[30] Kuo DJ, Lee YC, Huang WF (2007). Hormone therapy use and prescription durations of menopausal women in Taiwan: a 5 years’ National Cohort study. Maturitas.

[31] Sinclair M, O’Toole J, Malawaraarachchi M, Leder K (2012). Comparison of response rates and cost-effectiveness for a community-based survey: postal, internet and telephone modes with generic or personalised recruitment approaches. BMC Med Res Methodol.

